# Identification of a preferred DNA binding sequence and novel regulon member for CadR in *Pseudomonas aeruginosa* PAO1

**DOI:** 10.3389/fmicb.2025.1608957

**Published:** 2025-07-14

**Authors:** John K. Barrows, Ross M. Wood, Alaina B. Westee, Kamya A. Stubbs, Melanie Ratliff-Griffin, Michael W. Van Dyke

**Affiliations:** ^1^College of Humanities, Sciences, and Technology, Reinhardt University, Waleska, GA, United States; ^2^Department of Cellular and Molecular Biology, Kennesaw State University, Kennesaw, GA, United States; ^3^Department of Chemistry and Biochemistry, Kennesaw State University, Kennesaw, GA, United States

**Keywords:** gene regulation, CadR, transcription factors, metal-sensing, *Pseudomonas aeruginosa*

## Abstract

Transition metals, such as cadmium (Cd) and zinc (Zn), can be detrimental to cell viability in excess. Bacteria contain conserved regulatory mechanisms to sense and respond to a variety of environmental stressors, such as an influx of metal cations. One such mechanism is the activation of metalloregulatory transcription factors that bind to cognate metal cofactors to induce a transcription regulatory response. Metalloregulatory transcription factor families, such as the ferric uptake regulator (FUR), mercury-resistant regulator (MerR), copper-sensitive operon repressor (CsoR), and diphtheria toxin regulator (DtxR), are found widespread throughout bacterial genomes. Often, these transcription factors bind a specific DNA sequence found in the promoter of regulated genes to exert their transcription regulatory functions. In this study, we use an iterative selection technique called restriction endonuclease protection, selection, and amplification (RESPA) to identify the preferred DNA binding sequence for the MerR family, Cd/Zn-responsive regulator, CadR, from the opportunistic human pathogen, *Pseudomonas aeruginosa*. By doing so, we identify the transcription regulatory network for CadR, which includes the Cd/Zn-exporter, *cadA*, as well as an uncharacterized zinc ribbon domain-containing protein.

## 1 Introduction

The MerR family of transcription regulators is found extensively throughout bacteria. Structurally, these transcription factors contain a conserved *N*-terminal DNA-binding domain, a central dimerization helix, and a divergent C-terminal binding domain (Brown et al., 2003). MerR family members bind a diverse group of ligands, including a variety of heavy metal ions (Hg^+^, Zn^2+^, Cu^2+^, Cd^2+^, Pd^2+^, Co^2+^, and Ni^2+^), redox-responsive metabolites, and exogenous toxic compounds such as antibiotics (Fang and Zhang, 2022). Typically, promoters of MerR-regulated genes contain abnormally long spacer regions between the −35 and −10 elements, whereas the optimal spacing between these elements is 17+/−1 bp (Vassylyev et al., 2002). Apo-MerR dimers bind the DNA sequence between the −35 and −10 elements, often leading to transcriptional repression of the downstream gene. Upon effector binding, the MerR dimers distort DNA topology bringing the −35 and −10 elements in preferred proximity, thus allowing RNA polymerase holoenzyme recognition and subsequent transcription activation (Philips et al., 2015). Regulons for MerR transcription factors usually include efflux systems used to remove their often-toxic effector molecule.

Most bacterial transcription factors recognize a specific DNA sequence found immediately upstream of a regulated gene (Browning and Busby, 2004; Seshasayee et al., 2011). Several *in vitro* and *in vivo* techniques have been developed to identify preferred DNA binding sequences for transcription regulatory proteins, including chromatin immunoprecipitation (ChIP)-seq (Robertson et al., 2007), SELEX-seq, and high-density dsDNA microarrays (Wang et al., 2011). However, these technologies often need recombinant proteins with either *N*- or *C*-terminus tags, which may interfere with DNA binding. If a tag is not used for affinity capture, then antibodies are needed, which may not be readily available for bacterial proteins. An alternative, iterative selection approach is restriction endonuclease protection, selection, and amplification (REPSA; Barrows and Van Dyke, 2023). This technique is performed in sequential rounds, wherein in each round a population of preferred DNA-binding sequences is selected by type IIS restriction endonuclease (IISRE) digestion. DNA templates used in REPSA contain defined flanking regions harboring IISRE binding sites and an internal region of random nucleotides. IISREs bind DNA at a specific sequence and cleave DNA at a set distance away from where they bind. The DNA templates for REPSA are designed so that the IISRE cleaves DNA within the region of random nucleotides. If a transcription factor of interest binds DNA within the random region, IISRE-dependent cleavage will be blocked. Uncleaved DNAs are amplified by PCR, which are then used as inputs to the next round of REPSA. This process is repeated until an uncleaved DNA population is observed by gel electrophoresis, indicative of selecting high-affinity DNA binding sequences for the transcription factor of interest. Resulting DNAs are subject to high-throughput sequencing and motif discovery software to identify consensus DNA-binding motifs.

*Pseudomonas aeruginosa* is a gram-negative, ubiquitous bacterium commonly found in freshwater environments. *P. aeruginosa* is an opportunistic pathogen often infecting immunocompromised hosts, such as burn victims, cystic fibrosis patients, and those requiring treatment by ventilation (Qin et al., 2022). As such, *P. aeruginosa* is a common cause of nosocomial, or hospital-borne, infections and is considered a high-priority pathogen by the World Health Organization (Jesudason, 2024). Understanding the fundamental biology of *P. aeruginosa* has therefore become essential to laying the foundation for future treatment strategies.

In this study, we identify a preferred DNA binding motif for the cadmium (Cd^2+^)/zinc (Zn^2+^)-responsive MerR regulator, CadR, from *P. aeruginosa* PAO1. Similar to traditional MerR regulators, CadR homologs bind a specific DNA sequence and induce DNA distortion and transcription activation upon metal effector binding, as exemplified by the CadR homolog from *Pseudomonas putida* (Liu et al., 2019). Using REPSA, we find *Pa*CadR binds a palindromic, 23 bp inverted repeat containing 11 bp repeat units separated by 1 bp. When mapped to the *P. aeruginosa* PAO1 genome, we identified two sequences that exhibited high-affinity *Pa*CadR binding. Identification and regulation of genes adjacent to these binding sequences were validated *in vitro* and *in vivo*, leading to the promoter characterization of a previously unannotated open reading frame encoding a zinc ribbon domain-containing protein. Collectively, these results showcase the DNA specificity of a CadR homolog and provide a framework for transcription factor characterization in *P. aeruginosa.*

## 2 Materials and methods

### 2.1 *Pa*CadR protein expression and purification

A *P. aeruginosa* CadR expression vector was developed using GenScript^®^. To do so, the coding region of *Pa*CadR (*PA3689;* complement position 4,130,952–4,131,422 on *P. aeruginosa* PAO1 chromosome) as well as additional upstream sequences used for purification were codon optimized for expression in *E. coli* and inserted into a pET-11a expression vector using restriction enzyme sites, NdeI and BamHI. The complete amino acid sequence added before the *cadR* start codon was MGSSHHHHHHENLYFQGS, which includes a 6× histidine and TEV-protease cleavage sequence. *E. coli* strain Rosetta 2 (DE3) competent cells (Millipore) were transformed with the expression plasmid and selected for on agar plates containing 100 μg/mL ampicillin and 34 μg/mL chloramphenicol. Successfully transformed cells were initially grown in 2 mL Luria-Bertani (LB) broth at 37°C and 250 RPM for 1 h, then seeded into a 50 mL culture. The larger culture was grown at 37°C and 250 RPM to an OD of ~0.5 and then induced with 1 mM IPTG for 3 h. Cells were then pelleted and stored at −80°C. For purification, the pellet was thawed and resuspended in 1 mL 2X Bacterial Extract Buffer [40 mM Tris-Cl (pH 7.5), 200 mM NaCl, 0.2 mM EDTA, 5 mM βME and 1 mM PMSF]. Next, 22 μL of a 10 μg/μL lysozyme solution was added, and the mixture was incubated on ice for 10 min. The cell solution was then sonicated at 3 W, 10 s on/50 s off, for 5 cycles, and then pelleted. The resulting supernatant was supplemented with imidazole and NaCl to final concentrations of 20 mM and 500 mM, respectively, then loaded onto a His SpinTrap column (Cytiva) following the manufacturer’s protocol. Eluted material was combined, supplemented with 10 μL of 10 U/μL AcTEV protease (Invitrogen), and buffer exchanged to 50 mM Tris-Cl (pH 8.0), 0.5 mM EDTA, 1 mM βME by overnight dialysis at 4°C. The dialyzed sample was supplemented with imidazole and NaCl to final concentrations of 100 mM and 500 mM, respectively, then loaded onto a His SpinTrap column (Cytiva). Cleaved *Pa*CadR was collected from the flowthrough (Supplementary Figure S1), buffer exchanged to 2X Bacterial Extract Buffer, diluted 2-fold with 100% glycerol, and stored at −20°C.

### 2.2 DNA oligonucleotides

All DNA oligonucleotides used in this study are presented in Supplementary File S1. Each oligonucleotide was purchased from Integrated DNA Technologies (IDT) and purified using their standard desalting procedure. Selection template oligonucleotides for REPSA were amplified using ST5L and 5′ IRDye-700 labeled ST2R primers. Oligonucleotides used for biolayer interferometry (BLI) were amplified using ST2L and 5′ biotin-labeled ST2R primers. For electromobility shift assay (EMSA), the control DNA oligonucleotide was amplified using ST2R and 5′ IRDye-800 labeled ConL primers, while each experimental oligonucleotide was amplified with ST2L and 5′ IRDye-700 labeled ST2R primers. PCR reactions were performed using New England Biolabs (NEB) Taq DNA polymerase with standard Taq buffer under reaction conditions specified by the manufacturer. dsDNA quantification following PCR amplification was achieved using a Qubit 3 Fluorometer (Invitrogen).

### 2.3 Restriction endonuclease protection, selection, and amplification

REPSA was performed as described previously (Barrows and Van Dyke, 2023), with minor changes. Our selection template oligonucleotide (ST5R26, Supplementary File S1) contained defined regions flanking an internal 26-mer random nucleotide sequence. The random nucleotide region was created using IDT’s Handmix method with a 25% representation of each nucleotide. To add a 5′ IRDye700 label to our selection template, 200 ng of the ST5R26 oligonucleotide was subject to a single cycle of PCR using the 5′ IRDye700 labeled ST2R primer. The resulting DNAs were purified using a DNA Clean and Concentrator-5 kit (Zymo Research) and treated with 20 units Mung Bean Nuclease (NEB) in 1X CutSmart^®^ buffer (NEB) for 30 min at 37°C to remove ssDNA species. The resulting DNAs were purified with a DNA Clean and Concentrator-5 kit (Zymo Research) and subsequently used for the first round of REPSA. REPSA reactions were performed at 37°C in 1X CutSmart^®^ buffer containing 1 mM DTT, 0.1 ng/μL DNA template, and 100 nM *Pa*CadR, where indicated. After a 20-min incubation, type IIS restriction enzyme (IISRE) was added at 0.04 U/μL and mixed by thorough pipetting. Reactions were incubated for 5 more minutes at 37°C, then placed on ice to stop IISRE-cleavage. A 9 μL sample from reactions containing IISRE and *Pa*CadR was used for PCR amplification. The resulting DNAs were purified by a DNA Clean and Concentrator-5 kit (Zymo Research) and used as inputs for the next round of REPSA. The IISRE BpmI (NEB) was used for Rounds 1, 3, 5, 6, 8, and 9, while FokI (NEB) was used for Rounds 2, 4, 7, and 10.

### 2.4 DNA sequencing and bioinformatics

**Figure 1:**
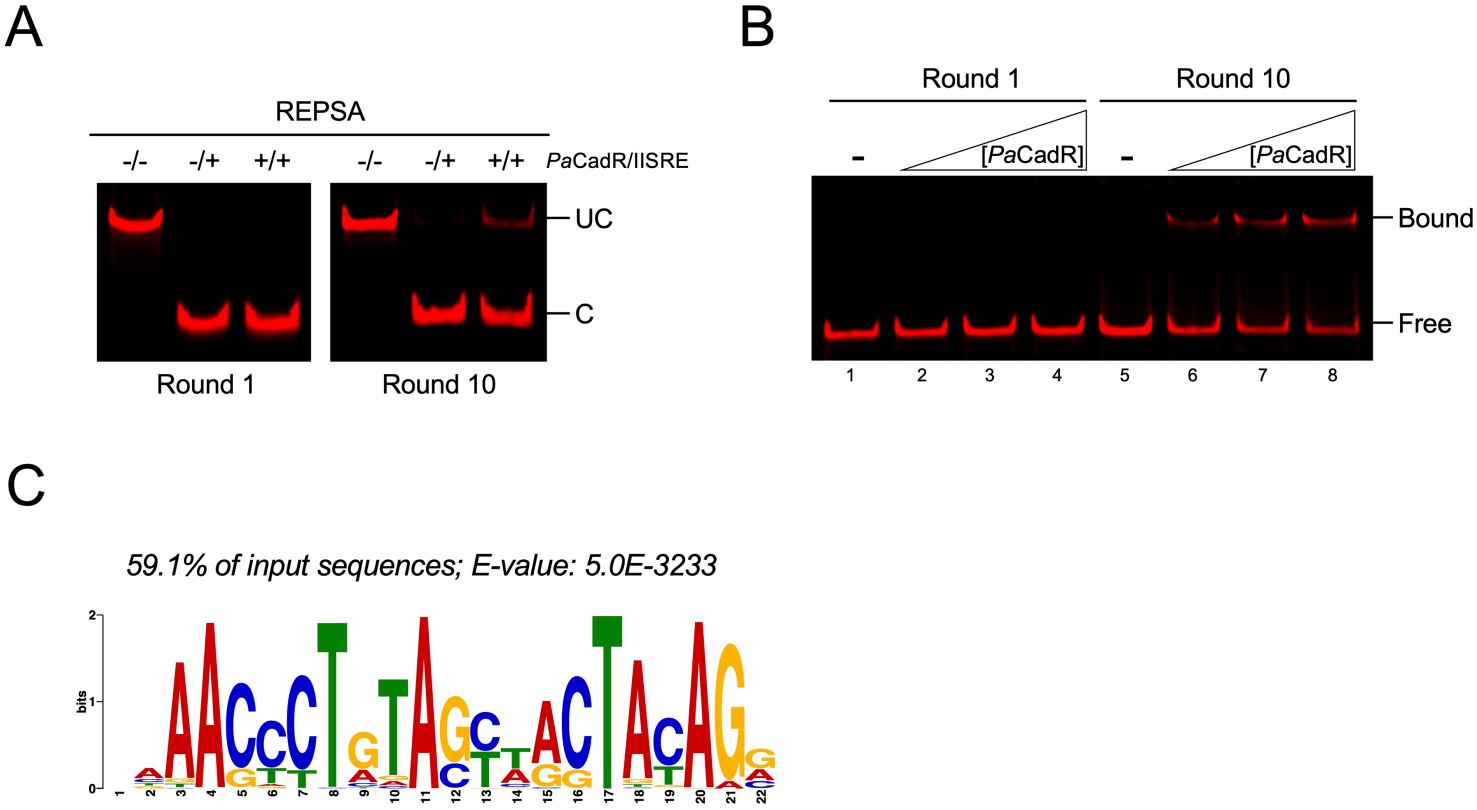
Identification of *Pa*CadR preferred DNA binding sequences by REPSA. **(A)** REPSA was performed with 100 nM *Pa*CadR. Results from Rounds 1 and 10 are shown. Uncut (UC) and cut (C) DNA bands are identified. Note the presence of an uncut DNA population in Round 10. **(B)** Input DNAs from Round 1 and Round 10 were incubated with 25, 50, or 100 nM *Pa*CadR. Samples were analyzed by native PAGE and visualized using a LICOR Odyssey imager. Protein-bound (Bound) and unbound (Free) DNA complexes are identified. **(C)** DNAs from Round 10 of REPSA were analyzed by high-throughput sequencing and motif elucidation software. The most common and significant DNA motif is presented.

DNAs from Round 10 of REPSA (Figure 1A) were subject to fusion PCR to add Nextera read sequences (“NT1/2”), Nextera XT index sequences (“2i”), and Illumina P5/P7 adapter sequences to each side of our DNA template (Supplementary File S1). The resulting DNAs were 183 nt in length. 75 pM barcoded DNAs and 50 pM phiX control v3 DNAs (Illumina) were analyzed by high-throughput sequencing using an iSeq100 system following the manufacturer’s instructions. Resulting fastq files were processed to remove defined flanking regions, leaving only the 26-mer randomized region. The refined sequencing library was input into Sensitive, Thorough, Rapid, Enriched Motif Elicitation (STREME) v 5.5.3^1^ to identify a consensus motif. Default STREME parameters were used, including shuffled input sequences as the control. The only deviation from the default parameters was the minimum motif width was set to 15, and the maximum motif width was set to 26. The position weight matrix in Supplementary Figure S2D was mapped to the *Pseudomonas aeruginosa* PAO1 VE13 uid 212,862 version 251 genome within the GenBank Bacteria Genomes and Proteins database using the Find Individual Motif Occurrences (FIMO) v 5.5.4.^2^ Default parameters were used for FIMO analysis, with a *p*-value cutoff of 1 × 10^−5^. Regulatory elements in gene promoters were predicted using Softberry BPROM (Solovyev and Salamov, 2011).^3^ Promoter prediction in *P. aeruginosa* was performed using SAPHIRRE. CNN.pseudmonas.^4^ Protein structure prediction was performed using the AlphaFold 3 server,^5^ and representative images were obtained using its default 3D visualization tool.

### 2.5 Biolayer interferometry

**Figure 2:**
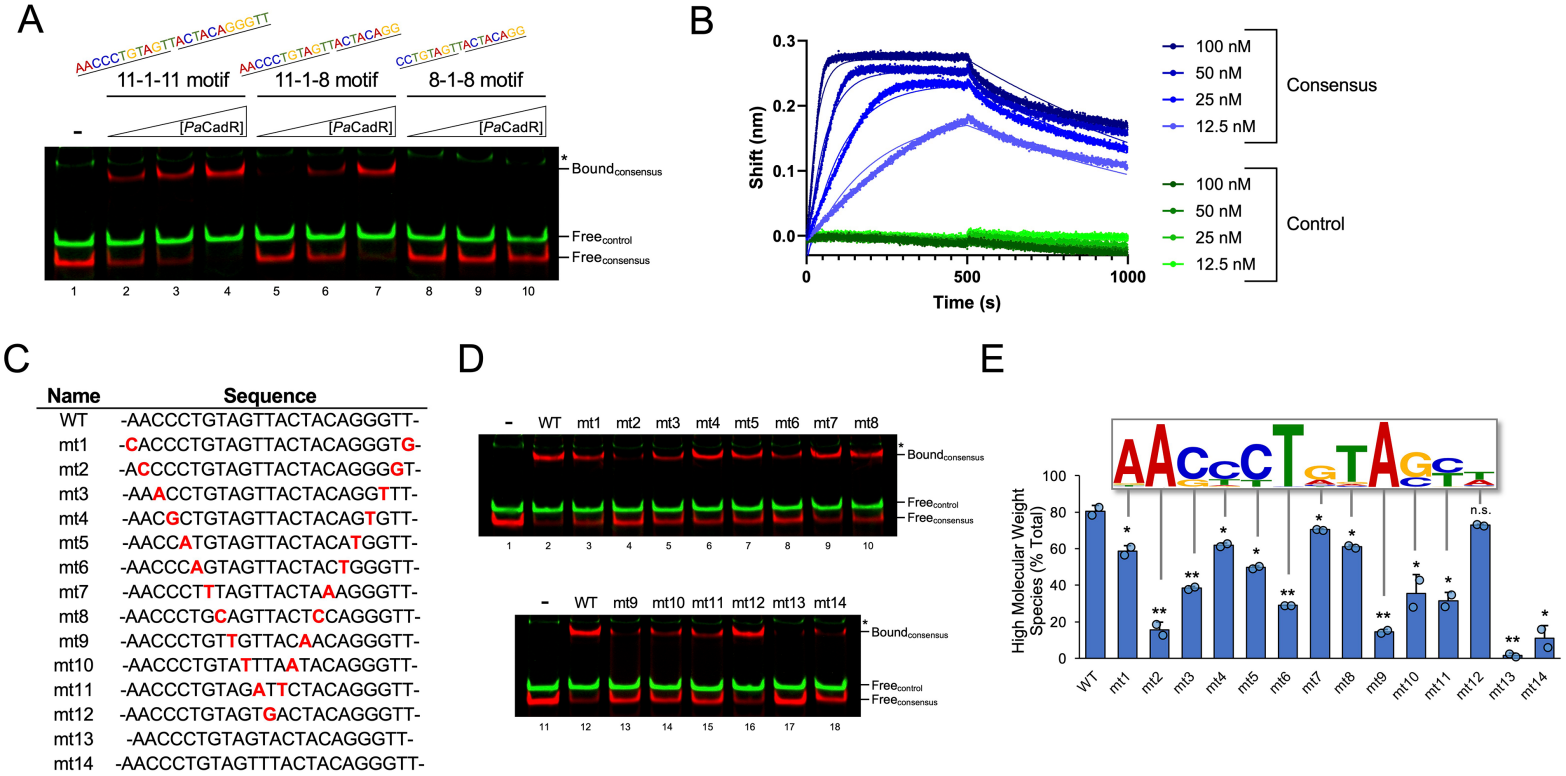
Mutational analysis of DNA binding sequence. **(A)** IRDye-700 labeled consensus sequence DNAs (red) and IRDye-800 labeled control DNA (green) were incubated with 20, 40, or 80 nM *Pa*CadR. Samples were analyzed by native PAGE and visualized using a LICOR Odyssey imager. Protein-bound (Bound) and unbound (Free) DNA complexes are identified. (*) Nonspecific IRDye-800 PCR product. **(B)** Biolayer interferometry was performed with 5′ biotin-labeled consensus DNA (blue traces) and control DNA (green traces). At time <500 s, DNA-loaded biosensors were subject to solutions containing the indicated concentrations of *Pa*CadR. At 500 s, biosensors were transferred to wells lacking *Pa*CadR. Kinetic values were determined using GraphPad Prism 9. **(C)** List of mutant DNAs used in **(D)**. Full-length oligos are presented in Supplementary File S1. Mutated nucleotides are bolded in red. Mutants 13 and 14 lack or add an additional nucleotide between the 11 bp inverted repeats, respectively. **(D)** IRDye-700 labeled consensus sequence DNAs (red) and IRDye-800 labeled control DNA (green) were incubated with 50 nM *Pa*CadR. Samples were analyzed by native PAGE and visualized using a LICOR Odyssey imager. Protein-bound (Bound) and unbound (Free) DNA complexes are identified. (*) Nonspecific IRDye-800 PCR product. **(E)** Quantification of protein-bound species in **(D)**. An overlay of one 11 bp inverted repeat from Figure 1C is presented. Each nucleotide position is aligned to its respective mutation. Error bars represent +1 standard deviation of two independent experiments. Student’s two-tailed *t*-test; *N* = 2; ^*^p < 0.05 and ^**^p < 0.005. Statistical analysis compared the binding of the indicated DNA template to the binding of the WT template.

BLI was performed essentially as described previously (Barrows and Van Dyke, 2022), with minor changes. All BLI experiments were conducted using an Octet Red96e system (FortéBio), streptavidin-coated Dip and Read Biosensors (FortéBio), and the following reaction buffer: 20 mM Tris-Cl (pH 7.5), 100 mM NaCl, 0.01% Tween-20, and 1 mM DTT. The experimental setup included the following steps in sequential order: an initial 100 s startup step where biosensors were incubated with buffer, a 900 s DNA loading step where biosensors were incubated with ~100 ng 5′ biotin-labeled DNA templates, a 100 s baseline step where biosensors were incubated in buffer, a 500 s association step where biosensors were incubated with the indicated amount of *Pa*CadR, and finally a 500 s dissociation step where biosensors were incubated in buffer. All steps were performed at 37°C. Shifts corresponding to association and dissociation steps were normalized to the shift observed at baseline immediately prior to association. These values were input into GraphPad Prism 9 and then graphed to create the curves shown in Figure 2B. *K*_d_ values were predicted using the nonlinear regression, association then dissociation model.

### 2.6 Electromobility shift assay

EMSA reactions were performed in 1X CutSmart^®^ buffer, 1 mM DTT, and 3 mM EDTA. Reactions contained 5 nM of each dsDNA template shown. To achieve the indicated concentration of *Pa*CadR, the stock *Pa*CadR solution was diluted with 1X CutSmart^®^ buffer and added at 1/10th reaction volume. Reactions were incubated at 37°C for 20 min, and then 6× EMSA dye (20% glucose, 0.9% Orange G) was added at 1/5th reaction volume. Samples were separated by 10% native PAGE (4 V/cm for 15 min, then 8 V/cm until the dye front reached the bottom of the gel) and visualized using a LI-COR Odyssey imaging system.

### 2.7 *Pseudomonas aeruginosa* PAO1 strains, growth, and gene expression analysis

Reference and Δ*cadR* strains of *P. aeruginosa* PAO1, as well as the replicative plasmid, pME6001, and a pME6001 derivative containing the *Pa*CadR promoter and coding region (pME6001-CadR), were generously gifted by Karl Perron, PhD (Ducret et al., 2020; University of Geneva, Geneva, Switzerland). To ectopically express a His-tagged CadR protein, we designed a gene fragment (GenScript^®^) containing ~260 bp upstream of *cadR* and the *Pa*CadR coding sequence, which included a -GSSHHHHHHSM- insertion after the initial methionine. The complete sequence is presented in Supplementary File S2, and it was cloned into pME6001 using BamHI and HindIII sites to create pME6001-HisCadR.

To create electrocompetent *P. aeruginosa* cells, strains were inoculated into 3 mL LB media in glass tubes and grown for 16 h at 37°C and 180 RPM, angled at ~45°. 1 mL of the culture was then pelleted and washed twice with 1 mL 300 mM D-sucrose. Cells were then resuspended in 100 μL 300 mM D-sucrose, and 100 ng pME6001 or its derivatives were electroporated into the indicated strain using a Gene Pulser Xcell (Bio-Rad) with pulse settings: 25 μF; 200 *Ω*; 2.5 kV. 1 mL of LB medium was added directly to the pulse cuvette, pipetted up and down, then the cells were transferred to a glass tube and shaken for 1 h at 37°C. Transformed cells were selected on LB-agar plates containing 50 μg/mL gentamicin.

To confirm *cadR* deletion in the Δ*cadR* strain, genomic DNA from reference and Δ*cadR* strains was isolated from streaked colonies using a Quick-DNA Fungal/Bacterial Miniprep kit (Zymo Research) following the manufacturer’s instructions. Resulting DNAs were amplified using the IVT_CadR/A_L1 and IVT_CadR/A_R1 primers to identify *cadR* and gPCR_fur_L and gPCR_fur_R primers to identify a genomic region upstream of *fur* (PA2384; Supplementary Figure S3).

For gene expression analysis, glycerol stocks of each experimental strain were streaked onto LB-agar plates and grown for 16 h at 37°C. For strains containing pME6001 or its derivatives, 50 μg/mL gentamicin was used in solid and liquid media. Single colonies were inoculated into 3 mL LB media in glass tubes and grown for 16 h at 37°C and 180 RPM, angled at ~45°. Where indicated, cultures were supplemented with 100 μM CdCl_2_ or 100 μM ZnSO_4_ for 10 min. Then, 1 mL of culture was pelleted and placed on ice. RNA from each sample was isolated within 1 h of cell pelleting. RNA isolation was performed using a Quick-RNA Fungal/Bacterial Miniprep kit (Zymo Research). The resulting nucleic acid was treated with 5 units DNase I (Zymo Research) for 15 min at room temperature, then purified using an RNA Clean & Concentrator kit (Zymo Research). qPCR reactions were performed using iTaq Universal SYBR Green One-Step Kit (Bio-Rad) in 10 μL reactions containing 20 ng purified RNA, 250 nM primers, 5 μL iTaq Universal SYBR Green Supermix, and 0.125 μL iScript Reverse Transcriptase. The thermocycler set up was as follows: 50°C, 10 min; 95°C, 1 min; (95°C, 10 s; 60°C, 30 s; fluorescent reading) × 39 cycles with an associated melt curve. Gene expression was normalized to the pyruvate dehydrogenase E1 component gene (*aceE*/*PA5015*). The efficiencies for each primer set used for qPCR are presented in Supplementary Figure S4.

### 2.8 Chromatin immunoprecipitation

Single colonies of Δ*cadR P. aeruginosa* PAO1 containing a His-CadR expression vector (pME6001-HisCadR) were inoculated into 35 mL LB containing 50 μg/mL gentamicin and grown at 37°C, 180 RPM for 16 h. 10 mL of the culture was pelleted, resuspended in 1 mL PBS (137 mM NaCl, 2.7 mM KCl, 10 mM Na_2_HPO_4_, and 1.8 mM KH_2_PO_4_) containing 1% formaldehyde, then rotated for 10 min at room temperature. 137 μL of 1 M glycine-NaOH (pH 7.5) was added, then the mixture was rotated for 30 min at 4°C. The cells were pelleted, washed twice with 1 mL IMAC Buffer (20 mM sodium phosphate, 300 mM NaCl, 10 mM imidazole, pH 7.4), and then resuspended in 1 mL IMAC Buffer. The cells were then sonicated at 2.5 W, 10 s on/50 s off. This was repeated five times; then, the mixture was centrifuged for 10 min at 16,000 × *g* and 4°C. The lysate was collected, and 10 μL was stored on ice as an INPUT sample. NEBExpress^®^ Ni-NTA Magnetic Beads (NEB) were obtained and gently vortexed to resuspend. 50 μL of the bead slurry was washed twice with 200 μL IMAC buffer and then added to the lysate. The mixture was rotated for 30 min at room temperature; then, the beads were washed thrice with 500 μL IMAC buffer. 50 μL of elution buffer (20 mM sodium phosphate, 300 mM NaCl, 500 mM imidazole, pH 7.4) was added to the beads and gently mixed for 2 min. 40 μL of elution buffer was added to the INPUT sample, and then both samples were incubated at 65°C for 16 h to reverse crosslinks. The resulting DNA was purified using the DNA Clean & Concentrator-5 kit (Zymo Research) and analyzed by qPCR.

### 2.9 *In vitro* transcription

Promoter templates were designed to include sequences ~250 nt upstream and ~250 nt downstream of the indicated translation start site and were amplified by PCR from the *P. aeruginosa* PAO1 genome (see Supplementary File S1 for primer sequences). The resulting templates were purified using a DNA Clean & Concentrator-5 kit (Zymo Research). *In vitro* transcription assays were performed in 1X *E. coli* RNAP reaction buffer (NEB) containing 0.3 mM NTPs, 100 nM promoter template, and 0.1 U/μL *E. coli* RNAP holoenzyme (NEB). Where indicated, reactions contained 4 μM *Pa*CadR and/or 100 μM CdCl_2_. Reactions were incubated at 37°C for 20 min, then 4 μL samples were treated with 0.5 units DNase I (Zymo Research) for 5 min at room temperature. Samples were then diluted 2-fold with 2X RNA Loading Dye (NEB). Prior to gel electrophoresis, samples were heated at 75°C for 5 min and then placed on ice for 2 min. Samples were separated by 5% TBE-urea PAGE (16 V/cm for 30 min) and visualized with SYBR Gold staining (Molecular Probes). To differentiate *cadR* and *cadA* RNA transcripts, templates of varying lengths were developed, and changes in output RNA length were determined (Supplementary Figure S5).

## 3 Results

### 3.1 Identification of the *Pa*CadR DNA binding sequence

Previous studies have characterized a single genomic binding site for CadR from *P. aeruginosa* PAO1 (Brocklehurst et al., 2003; Ducret et al., 2020; referred to hereafter as *Pa*CadR). However, whether *Pa*CadR binds multiple genomic locations is unknown. To uncover all potential genomic binding sequences, we first sought to identify a consensus DNA binding motif for *Pa*CadR using REPSA. Recombinant *Pa*CadR was purified using immobilized metal affinity chromatography, followed by TEV protease-dependent cleavage to remove a 6x histidine tag (Supplementary Figure S1). In the first round of REPSA, the addition of *Pa*CadR had no noticeable effect on the IISRE-dependent cleavage of selection template DNAs. However, by Round 10, we identified a cleavage-resistant population of DNA sequences that only occurred when incubated with *Pa*CadR (Figure 1A). The emergence of a protected DNA population in later rounds of REPSA suggests the identification of preferred *Pa*CadR binding sequences. To test the specificity of *Pa*CadR to our REPSA-identified DNAs, we performed an electromobility shift assay (EMSA) using DNAs from Round 1 or Round 10 of REPSA (Figure 1B). DNAs from Round 10 showed a *Pa*CadR-dependent shift that was not present with Round 1 DNAs, thereby confirming *Pa*CadR association.

To determine *Pa*CadR-specific DNA binding motifs, Round 10 DNAs were barcoded with sequences suitable for Illumina-based technologies (Nextera XT) and sequenced using an iSeq100 system. The resulting reads (153,845 total) were trimmed to include only the original internal cassette of random nucleotides from our selection template DNAs (26 nt in length). Motif elucidation was performed using Sensitive, Thorough, Rapid, Enriched Motif Elicitation (STREME) software from the MEME suite (Bailey et al., 2015). The most common and significant motif produced from this output, shown in Figure 1C, had a significance *E*-value of 5.0 × 10^−3233^ and was found in over 59% of input sequences. This motif can be described as pseudo-palindromic, containing a 17 bp inverted repeat with 8 bp repeat units from positions 6–13 and 15–22, which likely represents the preferred DNA binding motif for *Pa*CadR.

### 3.2 Validation and mutational analysis of the consensus DNA binding sequence

To validate *Pa*CadR binding to our REPSA-identified motif, we developed consensus DNA sequences containing the highest nucleotide count found at each position in the position weight matrix from Figure 1C. The presented motif is pseudo-palindromic, where positions 3–5 in the sequence motif contain nucleotides with high counts that are not reciprocated at the end of the motif (hypothetical positions 23–25). Therefore, we first tested the binding affinity of *Pa*CadR to a complete 11-1-11 palindromic sequence (AACCCTGTAGTTACTACAGGGTT), the 11-1-8 sequence present in the Figure 1C motif (AACCCTGTAGTTACTACAGG), and a minimal 8-1-8 palindromic sequence (CCTGTAGTTACTACAGG). To do so, we created DNA constructs containing each sequence and assayed *Pa*CadR binding by EMSA. *Pa*CadR bound to the 11-1-11 construct with the highest affinity yet still showed appreciable binding toward the 11-1-8 sequence (Figure 2A, red). However, incubation with the 8-1-8 sequence yielded little to no detectable binding. For each EMSA reaction, *Pa*CadR showed no apparent affinity to a control DNA that contained identical flanking regions as our consensus sequences (used for PCR amplification), yet a unique internal DNA sequence (Figure 2A, green). This result suggests *Pa*CadR preferentially binds a full palindromic sequence consisting of two 11 bp repeat units separated by one nucleotide.

When analyzing *Pa*CadR binding by EMSA, we observed a single shifted DNA band when complexed with *Pa*CadR (see “Bound” species in Figure 2A). Currently studied MerR homologs bind DNA as a homodimer to exert their transcription regulatory functions (Liu et al., 2019), although other bacterial metalloregulatory transcription factors have been shown to bind target DNA sequences as a dimer of dimers or higher-order oligomers (Baichoo and Helmann, 2002; Choi et al., 2024; Dwarakanath et al., 2012). To confirm the oligomer state of DNA-bound *Pa*CadR, we utilized an adaptation of a Ferguson plot (Orchard and May, 1993; Supplementary Figure S6). We found the apparent weight of DNA-bound *Pa*CadR to be ~38 kDa, which is consistent with dimeric binding (the estimated molecular weight of *Pa*CadR homodimer is ~36 kDa). Similarly, we found that purified *Pa*CadR exists predominantly as a homodimer in solution (Supplementary Figure S7).

To further analyze binding kinetics, we assayed *Pa*CadR binding to the 11-1-11 consensus sequence by biolayer interferometry (BLI). When consensus DNA sequences were probed with *Pa*CadR, we observed sensogram-type signal amplitudes (i.e., wavelength shifts) consistent with *Pa*CadR binding that were retained after extensive dilution (Time > 500 s; Figure 2B, blue traces). Kinetic analysis of this interaction yielded a dissociation constant, K_D_, of ~3 nM. DNA binding kinetics have not been performed for other CadR homologs; however, our low nanomolar K_D_ value is consistent with a previous finding for a MerR regulator (Shewchuk et al., 1989). When BLI was performed with DNAs containing a control sequence with an internal DNA sequence having no homology to the consensus sequence, we observed no apparent shift in wavelength (Figure 2B, green traces). Collectively, these data show high-affinity binding of *Pa*CadR to its REPSA-identified consensus DNA sequence.

The position weight matrix identified by STREME indicates the relative abundance of nucleotides at a certain position based on input sequences. Often, the more frequently a particular nucleotide is found in the same position, the more likely that nucleotide is critical for promoting a protein-DNA interaction. To formally address this, we created point mutations in each inverted repeat of the *Pa*CadR consensus motif (Figure 2C) and assayed binding by EMSA (Figure 2D). Each mutation contained the least common nucleotide found at that position based on the position weight matrix. Every mutation occurring within the 11 bp repeat regions significantly reduced *Pa*CadR binding compared to the wild-type (WT) consensus sequence at the tested *Pa*CadR concentration (Figures 2D,E). As expected, the mutations within this region that affected binding the most occurred at positions containing the highest single nucleotide counts (Figure 2E, mts 2, 6, and 9). Removing the linker bp between the repeat regions (mt13) or adding an additional linker bp (mt14) also significantly reduced *Pa*CadR binding. The only mutation that did not significantly alter *Pa*CadR binding was mutating the linker nucleotide itself, even though the position weight matrix from REPSA suggested either a T or A preference at that position (Figure 1C, position 14; Figure 2E, mt 12). Altogether, these data suggest that the position weight matrix in Figure 1C accurately models the relative importance of each nucleotide in promoting a DNA-*Pa*CadR interaction.

### 3.3 Identification and validation of genomic binding sequences

To identify potential genomic *Pa*CadR binding sequences, we input the position weight matrix from the *Pa*CadR consensus binding motif into Find Individual Motif Occurrences (FIMO) software (Grant et al., 2011) to scan the *P. aeruginosa* PAO1 genome. To create a complete position weight matrix for the 11-1-11 motif, we supplemented the 8 bp repeat unit from the STREME output in Figure 1C with the inverse of the three additional nucleotide counts from the full 11 bp repeat unit (Supplementary Figure S2). The output of the FIMO analysis with a cutoff significance *E*-value of 6 × 10^−7^ is presented in Table 1. Two genomic sequences significantly matched the input motif with a *p* < 1 × 10^−9^. As expected, one sequence (gDNA1_cadA/R) was found in the promoter region of the Cd^2+^/Zn^2+^-specific exporter, *cadA* (*PA3690*), which is regulated by *Pa*CadR (Brocklehurst et al., 2003; Ducret et al., 2020). The other sequence (gDNA2) was not found within −200/+20 bp of a transcription start site, the most common region to find binding sites for bacterial transcription factors. The next most significant sequences had *p*-values over two orders of magnitude lower than the previous and were mostly found over 200 bp away from transcription start sites.

**Table 1:** FIMO analysis of REPSA-identified motif.

Start position	Stop position	Within −200/+20 bp of TSS?	Gene(s)	Strand	*p*	*q*	Matched sequence	Sequence name
4,131,470	4,131,492	Yes	*cadA/cadR*	−	2.57 × 10^−10^	0.0026	CACCCTGTAGCAACTACAGAGTC	gDNA1_cadA/R
4,131,470	4,131,492	Yes	*cadA/cadR*	+	6.50 × 10^−10^	0.0026	GACTCTGTAGTTGCTACAGGGTG	—
1,112,233	1,112,255	No		+	7.41 × 10^−10^	0.0026	GACCCTGTAGTGGCTACAGCCTT	gDNA2
1,112,233	1,112,255	No		−	8.26 × 10^−10^	0.0026	AAGGCTGTAGCCACTACAGGGTC	—
5,252,289	5,252,311	No		−	2.12 × 10^−7^	0.531	AGCTCTACAGCAACTACAGCTTC	gDNA3
5,935,282	5,935,304	No		−	4.47 × 10^−7^	0.627	GTCCTTGTAGGCAGTATAGGGAT	gDNA4
5,252,289	5,252,311	Yes	*marR*	+	4.82 × 10^−7^	0.627	GAATCCGTAGTCGCTCCAGCGTT	gDNA5_marR
3,950,000	3,950,022	No		+	4.91 × 10^−7^	0.627	GAAGCTGTAGTTGCTGTAGAGCT	—
4,969,207	4,969,229	No		+	5.05 × 10^−7^	0.627	AACCCTCAACGACCTGCAGAGTG	gDNA6
284,726	284,748	Yes	*marR*	−	5.22 × 10^−7^	0.627	AACGCTGGAGCGACTACGGATTC	—
284,726	284,748	No		+	5.51 × 10^−7^	0.627	TACCCTCTACGAAGTACAGCGCA	gDNA7

A position weight matrix for the 11-1-11 PaCadR consensus DNA binding motif was analyzed using FIMO. Genomic regions with a *p*-value < 6 × 10^−7^ are presented. Sequences found within −200 bp or +20 bp of a transcription start site (TSS) are indicated, as well as the corresponding nearby gene(s). (*p*-value) The probability of a random sequence of similar length matching the input motif with an as good or better score. (*q*-value) False discovery rate if the identified sequence is accepted as significant. (Start/stop position) Nucleotide sequence in the *P. aeruginosa* PAO1 genome (GenBank: CP006831.1) where the indicated sequence begins and ends. (Sequence Name) Assigned name for the indicated sequence. Sequences containing the complete complement of a previous sequence were not given annotations.

**Figure 3:**
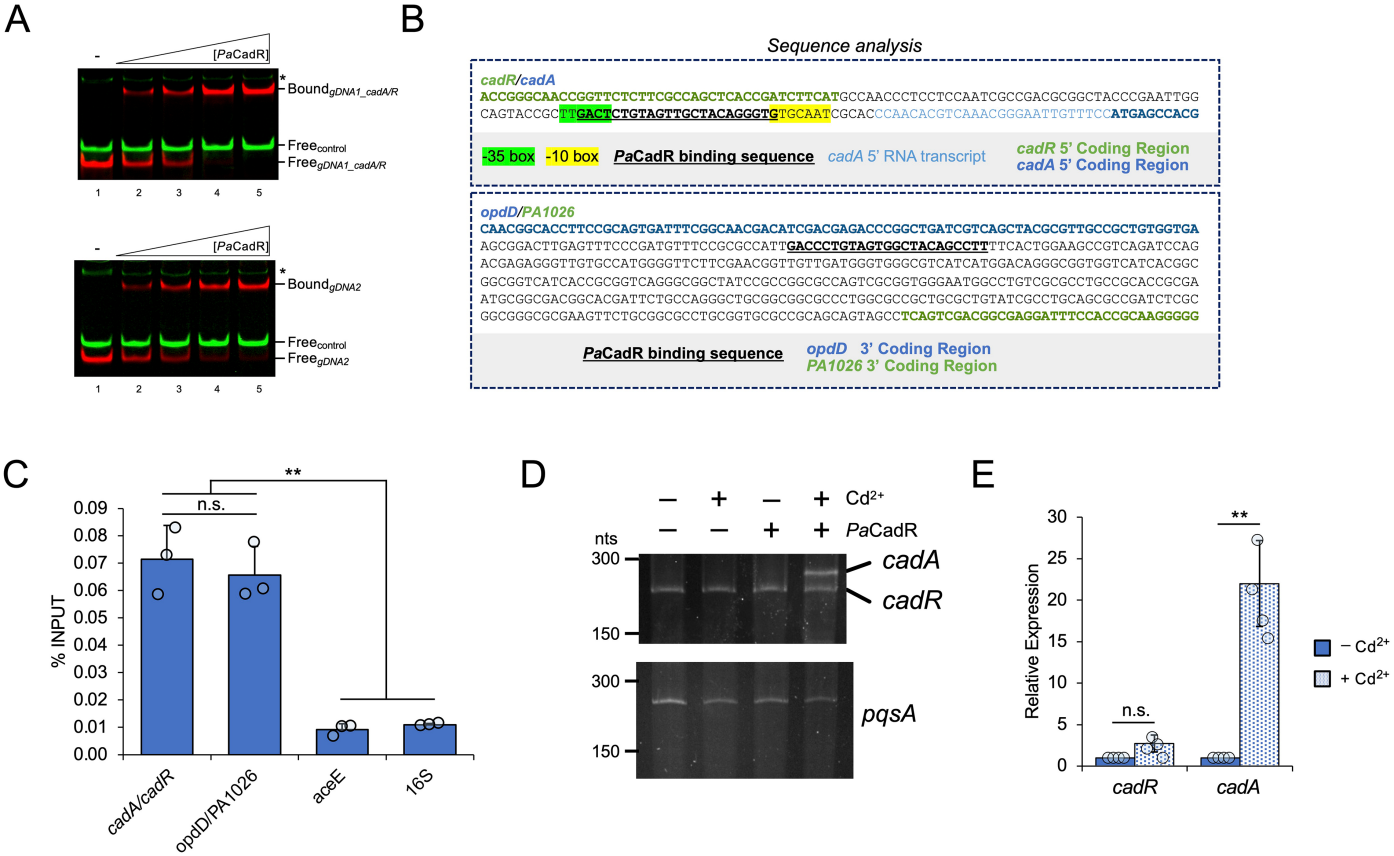
Validation of genomic binding sequences and *in vitro* analysis of *cadR* autoregulation. **(A)** IRDye-700 labeled genomic DNA (red) and IRDye-800 labeled control DNA (green) were incubated with 20, 40, 80, or 160 nM *Pa*CadR. Samples were analyzed by native PAGE and visualized using a LICOR Odyssey imager. Protein-bound (Bound) and unbound (Free) DNA complexes are identified. (*) Nonspecific IRDye-800 PCR product. **(B)** Analysis of genomic regions containing *Pa*CadR binding sequences. −10, −35, and transcription start sequences were predicted using SoftBerry BPROM. **(C)** DNA regions bound to ectopically expressed, His-tagged *Pa*CadR were purified by ChIP and analyzed by qPCR. Recovered DNAs are presented as a percentage of input DNAs, which were not subject to metal affinity chromatography. Error bars represent +1 standard deviation of three independent experiments. Student’s two-tailed *t*-test; *p* > 0.2 (n.s.), ^**^p < 0.005. **(D)** *In vitro* transcription reactions were performed containing 0.1 U/μL *E. coli* RNA polymerase holoenzyme, 100 nM of the *cadA/cadR* or *pqsA* promoter template, and 4 μM *Pa*CadR or buffer control. Where specified, reactions contained 100 μM CdCl_2_. Samples were treated with DNase I and then separated by denaturing PAGE. RNA was visualized by SYBR Gold staining. **(E)** RNA was isolated from *P. aeruginosa* PAO1 before and after a 10-min treatment with 100 μM CdCl_2_. Gene expression for *cadR* and *cadA* was quantified by RT-qPCR and normalized to the expression of *aceE* (pyruvate dehydrogenase E1 component, *PA5015*). Values are presented relative to expression prior to CdCl_2_ addition. Error bars represent +/− 1 standard deviation between four independent experiments. Student’s two-tailed *t*-test with unequal variance; *p* > 0.05 (n.s.), ^**^p < 0.005.

To validate *Pa*CadR binding to these genomic regions *in vitro*, we developed DNA templates that contained each genomic sequence presented in Table 1 and assayed *Pa*CadR binding by EMSA. We observed high affinity binding to the two most significant, FIMO-identified, genomic sequences (gDNA1_cadA/R and gDNA2; Figure 3A), which was comparable to *Pa*CadR binding to its REPSA-identified consensus sequence (compare Figures 2A, 3A). Conversely, *Pa*CadR exhibited little to no affinity toward the remaining DNAs from Table 1 (Supplementary Figure S8). This result identifies two genomic binding sequences for *Pa*CadR and highlights its apparent sequence specificity.

To further study the *in vitro*-validated *Pa*CadR genomic binding sequences, we analyzed each sequence in relation to nearby genes and predicted promoter elements (Figure 3B). The previously established *Pa*CadR binding sequence in the *cadA* promoter is positioned in between the −35 and −10 elements, typical of a MerR regulator. In *P. aeruginosa*, the *cadR* gene is found upstream of the *cadA* gene, transcribed in the opposite direction. No obvious promoter elements were identified for the *cadR* gene using prediction software, and whether *Pa*CadR regulates its own promoter has not been formally addressed. The second *Pa*CadR binding sequence was found within a 376 bp, intergenic region between converging genes, *opdD* (*PA1025*) and an uncharacterized transcription elongation factor (*PA1026*). To validate *Pa*CadR binding to these genomic sequences *in vivo*, we performed ChIP using *P. aeruginosa* PAO1 strains expressing a 6x-histidine tagged CadR construct (Supplementary Figure S9). When compared to control DNA regions (*aceE* and *16S rRNA* genes), we observed significantly increased recovery of DNA containing the *cadR/cadA* promoter as well as the intergenic region between *opdD* and *PA1026* (Figure 3C). Collectively, this data validates *Pa*CadR-binding to two genomic sequences *in vivo*.

### 3.4 *Pa*CadR does not regulate its own promoter *in vitro*

*Pa*CadR-dependent regulation of *cadA* has been shown previously (Brocklehurst et al., 2003; Ducret et al., 2020). However, autoregulation of *cadR* has not been extensively studied. To address this, we developed an *in vitro* transcription assay using *E. coli* RNA polymerase holoenzyme and a DNA template containing 5′ coding and promoter regions for *P. aeruginosa* PAO1 *cadR* and *cadA*. DNA templates with different lengths were initially used to differentiate between *cadR* and *cadA* transcripts (Supplementary Figures S5A,B). A control sequence containing the promoter and 5′ coding region from *pqsA* was also used. RNA expression from the *pqsA* promoter occurred regardless of Cd^2+^ or *Pa*CadR addition (Figure 3D). As expected, expression from the *cadA* promoter dramatically increased when both *Pa*CadR and Cd^2+^ were added to the reaction (Figure 3D). Conversely, the expression of *cadR* was not altered with the addition of *Pa*CadR and Cd^2+^. This result demonstrates *Pa*CadR-dependent transcription activation *in vitro* and suggests *Pa*CadR does not regulate gene expression from its own promoter. Furthermore, when cultures of *P. aeruginosa* PAO1 were treated with Cd^2+^, expression of *cadA*, but not *cadR*, increased significantly (Figure 3E). Together, these data suggest *Pa*CadR does not autoregulate *cadR* expression.

### 3.5 Regulation of an uncharacterized zinc ribbon domain-containing protein transcript

**Figure 4:**
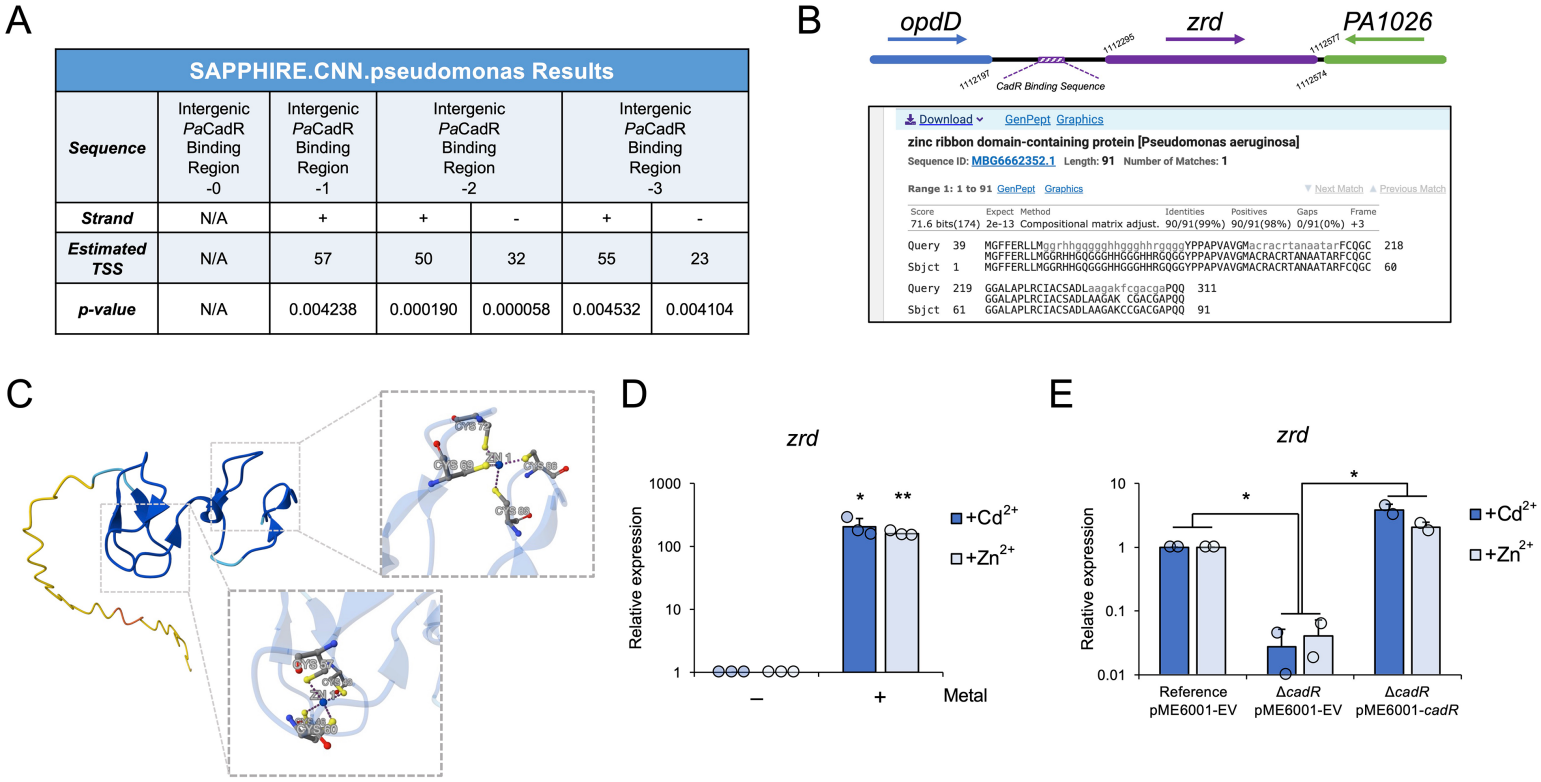
*Pa*CadR activates the expression of an uncharacterized transcript. **(A)** A 60 nt region containing the *Pa*CadR binding sequence between converging genes, *opdD* and *PA1026*, was analyzed by SAPPHIRE promoter prediction software. Where indicated, one, two, or three nucleotides from the center of the *Pa*CadR binding sequence were removed. (N/A) no promoter elements with a *p* < 0.05 were found. (Strand) promoter sequences were found on submitted DNA sequence (+) or complementary sequence (−). (Estimated TSS) the predicted transcription start site of the indicated promoter. **(B)** (Top) A model depicting the genomic location of *zrd* in relation to *opdD* and *PA1026*. Nucleotide positions correspond to the *P. aeruginosa* PAO1 genome. (Bottom) blastx result from a nucleotide query containing the region between the *Pa*CadR binding sequence and PA1026 coding region. **(C)** AlphaFold 3 output for the amino acid sequence shown in **(B)**, as well as two zinc ions. Residues involved in zinc coordination are shown in detail. **(D)** RNA was isolated from *P. aeruginosa* PAO1 before and after a 10-min treatment with 100 μM CdCl_2_ or 100 μM ZnSO_4_. Gene expression for *zrd* was quantified by RT-qPCR and normalized to the expression of *aceE* (pyruvate dehydrogenase E1 component, *PA5015*). Values are relative to the expression before metal addition. Statistical analysis compared the indicated sample to the sample before metal addition. Error bars represent one standard deviation between three independent experiments. Student’s two-tailed *t*-test with unequal variance: ^*^p < 0.05 and ^**^p < 0.005. **(E)** RNA was isolated from the indicated *P. aeruginosa* PAO1 strains after a 10-min treatment with either 100 μM CdCl_2_ or 100 μM ZnSO_4_. Gene expression for *zrd* was quantified by RT-qPCR and normalized to the expression of *aceE* (pyruvate dehydrogenase E1 component, *PA5015*). Values are relative to the reference strain containing the empty pME6001 vector (pME6001-EV). Error bars represent one standard deviation between two independent experiments. When comparing the Reference pME6001-EV strain to the Δ*cadR* pME6001-EV strain, a Student’s two-tailed *t*-test with unequal variance was used. When comparing the Δ*cadR* pME6001-EV strain to the Δ*cadR* pME6001-CadR strain, a Student’s two-tailed *t*-test with equal variance was used. ^*^p < 0.05.

Since MerR regulators often bind promoter regions, we were surprised to identify a *Pa*CadR binding site within an intergenic region between two converging genes (Figure 3B). We postulated that a previously uncharacterized promoter may be present in this region. MerR regulators can activate the expression of genes whose promoters contain regulatory elements (i.e., the −10 and −35 sequences) that are spaced too far apart for recognition by RNA polymerase holoenzyme. MerR binding distorts the DNA double helix and pulls the recognition sequences closer together, thus leading to gene expression. This distortion can be mimicked *in vitro* by removing nucleotides between these promoter recognition sequences (Parkhill and Brown, 1990). To test whether *Pa*CadR may activate a dormant promoter within the *opdD* and *PA1026* intergenic region, we input a 60 nt region containing the *Pa*CadR binding sequence into *Pseudomonas* promoter prediction software (Coppens and Lavigne, 2020; Figure 4A). As expected, the unaltered sequence did not identify a *Pseudomonas* promoter. However, as we removed nucleotides from the middle of the *Pa*CadR binding sequence, thus mimicking CadR-dependent DNA distortion, promoter sequences were identified. The most significant promoter sequence was identified when two nucleotides were removed from the *Pa*CadR binding sequence. Similar results were observed when a promoter analysis was conducted using the *cadA* promoter sequence (Supplementary Figure S10). This result suggests the presence of a non-coding RNA or an open reading frame within the *opdD* and *PA1026* genes.

To identify a potential open reading frame in this region, we analyzed the intergenic DNA sequence between the *Pa*CadR binding sequence and *PA1026* by BLAST (blastx; Figure 4B). The results identified an open reading frame encoding a zinc ribbon domain-containing protein (referred to hereafter as “Zrd”) that has been identified in more recent annotations of *P. aeruginosa* genomes but was not included in the *P. aeruginosa* PAO1 annotation. The coding region of this potential protein product is found downstream of the *Pa*CadR DNA binding sequence, suggesting *Pa*CadR regulates its expression (Figure 4B). Zinc ribbons, a subgroup of zinc finger domains, are found throughout eukaryotic and prokaryotic proteins and are often identified in nucleic acid-binding proteins (Krishna et al., 2003; Malgieri et al., 2015). *zrd* exhibits two zinc ribbon motifs (CXXC-N_10_-CXXC) likely allowing for coordination of two metal ions per protein monomer. To analyze the structure of the potential Zrd protein product, we used AlphaFold 3 (Abramson et al., 2024). This predicted structure shows a disordered N-terminus region along with an ordered C-terminus that contains two, Cys4 metal coordination sites, consistent with dual zinc ribbon domains (Figure 4C). To our knowledge, the *zrd* open reading frame has only been predicted computationally, and no experimental work on this genomic region has been conducted in *P. aeruginosa*.

We sought to determine if *zrd* expression was controlled by *Pa*CadR. First, we found that *zrd* was activated in response to both Cd^2+^ and Zn^2+^ *in vivo* (Figure 4D), consistent with CadR-regulated genes (Brocklehurst et al., 2003; Ducret et al., 2020). To formally implicate *Pa*CadR in *zrd* regulation, we analyzed gene expression in response to Cd^2+^ or Zn^2+^ in the reference and Δ*cadR P. aeruginosa* PAO1 strains containing different plasmids. In response to either metal, reference strains exhibited significantly higher expression levels of *zrd* compared to Δ*cadR* strains (Figure 4E). Activation of *zrd* was completely rescued when Δ*cadR* strains were supplemented with an expression plasmid containing the *Pa*CadR coding sequence (Figure 4E). As a positive control, we observed similar results for *cadA* expression (Supplementary Figure S11A), and the reconstituted expression of *cadR* was validated by qPCR (Supplementary Figure S11B). These results show that the increased expression of *zrd* in response to Cd^2+^ or Zn^2+^ is controlled by *Pa*CadR, thus adding a new member to the *Pa*CadR regulon. Together, these findings provide an initial characterization of a new metal-responsive open reading frame in *P. aeruginosa*.

## 4 Discussion

In this study, we identified the preferred DNA binding sequence for a CadR homolog in *P. aeruginosa* PAO1 using the *in vitro* iterative selection approach, REPSA (Figures 1A–C). REPSA can be performed with untagged, recombinant transcription factors where the DNA-protein interaction does not have to survive affinity purification or gel electrophoresis, thus providing several advantages to other iterative selection approaches. Indeed, a previous study was unsuccessful in identifying a preferred DNA binding sequence of 6xHis-tagged *Pa*CadR by high throughput-SELEX, albeit the researchers only conducted 4 rounds of selection (Wang et al., 2021). Here, we observed the successful selection of *Pa*CadR binding sequences by Round 10 of REPSA. Using high throughput sequencing, motif elucidation, and *in vitro* binding validation, we found *Pa*CadR preferentially binds a 23 bp inverted repeat consisting of 11 bp repeat units separated by 1 bp. Our 11-1-11 binding motif shows strong similarities, as well as minor differences, to the predicted CadR binding motif from the *Pseudomonadaceae* family in RegPrecise (Novichkov et al., 2013),^6^ which is built using predicted and validated genomic binding sequences. The remarkable similarities between these motifs highlight REPSA as a legitimate tool for determining preferred DNA binding sequences, while the differences potentially underscore genus- or species-specific DNA binding preference. Notably, the DNA binding specificity of CadR homologs seems to be evolutionarily conserved throughout Pseudomonadota, as a similar 10-1-10 binding motif has been predicted for CadR homologs in Alphaproteobacteria, Betaproteobacteria and Gammaproteobacteria (Supplementary Figure S12).

By mapping our 11-1-11 consensus DNA binding motif to the *P. aeruginosa* PAO1 genome, we identified two genomic sequences that *Pa*CadR bound with high affinity (Table 1). One of these sequences was found upstream of the Cd^2+^/Zn^2+^-specific exporter, *cadA*, for which CadR-dependent regulation has been validated previously (Brocklehurst et al., 2003; Ducret et al., 2020). However, *cadA* and *cadR* are divergent genes, and autoregulation of *cadR* has not been thoroughly studied in *P. aeruginosa*. Using an *in vitro* transcription system, we show expression of *cadA*, but not *cadR*, was specifically activated in the presence of Cd^2+^ and *Pa*CadR (Figure 3D). We also show expression of *cadR* is not activated by Cd^2+^ *in vivo* and previous studies have shown expression of *cadR* is not activated by Zn^2+^ (Ducret et al., 2020), further discounting autoregulation of *cadR*.

A second *Pa*CadR binding sequence was found within an intergenic region between two converging genes. Using promoter prediction software and BLAST, we identified an uncharacterized open reading frame in this region that is controlled by *Pa*CadR. This gene is predicted to encode a small protein containing two zinc ribbon domains, which we have called Zrd. We show transcription of *zrd* is induced by either Zn^2+^ or Cd^2+^ *in vivo*, and this activation is mediated by *Pa*CadR (Figures 4D,E). AlphaFold prediction of Zrd structure showed a highly disordered *N*-terminus region (Figure 4C). While intrinsically disordered domains can have important biological functions, we cannot rule out the use of a downstream translation start site that only translates the zinc ribbon domains.

Based on the structural prediction and gene expression results, Zrd exhibits several similarities to the metallothionein family of proteins. Metallothioneins are found throughout all domains of life and include small, cysteine-rich proteins that function in sequestering metal ions, thereby protecting the cell from heavy metal toxicity (Yang et al., 2024). Bacterial metallothioneins often contain at least one polynuclear metal cluster, as exemplified by SmtA (Blindauer et al., 2001), whereas Zrd is predicted to contain only mononuclear metal-binding sites (Figure 4C). A similar polynuclear cluster to SmtA is shown in *Pseudomonas* metallothioneins (Habjanič et al., 2020), and a previously characterized metallothionein in *P. aeruginosa*, PmtA, appears to play a role in biofilm formation and protecting against oxidative stress (Thees et al., 2021). Given *zrd* activation in response to excess Zn^2+^ or Cd^2+^, a plausible model is that metal-bound CadR activates *zrd* expression leading to Zn^2+^/Cd^2+^ coordination through Zrd’s two Cys4 metal-binding sites. Metal coordination by Zrd could function as a mode of sequestration, thereby reducing the intracellular pool of free Zn^2+^/Cd^2+^ ions. This proposed mechanism, in conjunction with increased Zn^2+^/Cd^2+^ export through CadR-dependent activation of *cadA* expression, would likely help alleviate metal toxicity. Although this study uncovers the genetic regulation of *zrd*, future work will help establish the biological function of its protein product and determine how Zrd contributes to metal homeostasis in *P. aeruginosa*.

## Data Availability

The original contributions presented in the study are included in the article/Supplementary material, further inquiries can be directed to the corresponding author.

## Footnotes

^1^ https://meme-suite.org/meme/tools/streme

^2^ https://meme-suite.org/meme/tools/fimo

^3^ https://www.softberry.com/berry.phtml?topic=bprom&group=programs&subgroup=gfindb

^4^ https://sapphire.biw.kuleuven.be/

^5^ https://alphafoldserver.com/

^6^ https://regprecise.lbl.gov/regulog.jsp?regulog_id=5201
