# Supplementary material for: Identification of a preferred DNA binding sequence and novel regulon member for CadR in Pseudomonas aeruginosa PAO1
Source: Front Microbiol. 2025 Jul 14;16:1608957. doi: 10.3389/fmicb.2025.1608957 (PMC12301328; doi:10.3389/fmicb.2025.1608957)
Supplement: Supplementary file 2 [file Table_2.DOCX]

>His-CadR sequence used for cloning into pME6001 at BamHI and HindIII sites

AGAGCCAAGCTTTCCATCGCTTCGATGCGCAGGTCGCTCCAGCGCAACTCGGCGTTGCCGGCCAGGGTTCCGATGGCGGCCACCGCCGGGCGAGGCGCGTGGCGGTGCCCATGATCATGTCCGTGACCGTGACCGTGACAGCAAGTGTCGGCGTGTTCGTGGCTCATGGAAACAATTCCCGTTTGACGTGTTGGGTGCGATTGCACACCCTGTAGCAACTACAGAGTCAAGCGGTACTGCCAATTCGGGTAGCCGCGTCGGCGATTGGAGGAGGGTTGGCATGGGAAGTTCACATCACCATCACCACCACAGCATGAAGATCGGTGAGCTGGCGAAGAGAACCGGTTGCCCGGTGGAGACCATCCGCTACTACGAGCGCGAAGGCCTGTTGCCCGAGCCCGCGCGTAGCGAAGGCAACTATCGGCAATACACCCTGGCGCATGTCGAGCGCCTGTCGTTCATCCGTCACTGCCGCTCGCTGGACATGACCCAGGAGGAAATCCGTACCCTGCTGGCGTTGCGCGACCGTCCCGAGGCGGATTGCGGCACCGCCAACCGGTTGATCGACGAGCACCTGCATCACGTCGAGGTGCGCATCGCCGAACTCCAGGCATTGCGCGAGCAACTGCGGGATCTCGGCTCACGCTGTACGGTCGCCGGCAACAGCCAGGCCTGCGGCATCCTCCGCGAACTGGAGCAGCCCGCGCCGCTGTCGCCAATCGCCGAGGAATGCGCCGAGGCCGGGCACATGCACGTCCCCGGCGTGCACCGCCGGCATGGCTGAAGCCGCGCTCAGGGATCCGGCTCT
