# Supplementary material for: Identification of a preferred DNA binding sequence and novel regulon member for CadR in Pseudomonas aeruginosa PAO1
Source: Front Microbiol. 2025 Jul 14;16:1608957. doi: 10.3389/fmicb.2025.1608957 (PMC12301328; doi:10.3389/fmicb.2025.1608957)
Supplement: Supplementary file 3 [file Data_Sheet_1.PDF]

Supplementary Material

1 Supplementary Data

1.1 Supplementary Files

Supplementary File S1. List of plasmids and oligonucleotides used in this study.

Supplementary File S2. The His-CadR sequence that was used for cloning into pME6001 at its BamHI and HindIII sites.

2 Supplementary Figures and Tables

2.1 Supplementary Figures

Supplementary Figure S1. *Pa*CadR purification. *E. coli* Rosetta 2 (DE3) cells were transformed with an expression vector containing a 6x histidine-tagged *Pa*CadR construct. Cell lysates were subject to His SpinTrap (Cytiva) purification and eluted fractions were combined (lane 4) and treated with TEV protease (lane 5). The resulting sample was again added to a His SpinTrap (Cytiva) where flow-through samples (lanes 6 and 7) were collected for long-term storage. (MW), molecular weight marker.

Supplementary Figure S2. Position weight matrices from STREME output and FIMO input. **(A)** The consensus motif output from STREME is presented. An 8 bp inverted repeat is labeled (positions 15 – 22) as well as a potential 11 bp inverted repeat (positions 3 – 13). **(B)** To create a matched 11-1-11 consensus motif, the reverse complement of nucleotides found in positions 3 – 5 should be added to positions 23 – 25. **(C)** Position weight matrix for the motif in (*A*) from positions 3 – 22. **(D)** Position weight matrix for the motif in (*A*) from positions 3 – 22 and the reverse complement of the nucleotides in positions 3 – 5 at positions 23 – 25

Supplementary Figure S3. Genomic PCR to confirm *cadR* deletion. Genomic DNA was isolated from reference and Δ*cadR* strains of *P. aeruginosa* PAO1. DNA samples were subject to 30 cycles of PCR using primers specific for the *cadR* or *fur* (*PA2384; "*Control gene") promoter. Primer sequences can be found in **Supplementary File S1**. PCR products were analyzed by 1% agarose gel electrophorese and visualized by ethidium bromide staining. (MW), molecular weight marker.

Supplementary Figure S4. Quality control for qPCR primers. **(A – G)** Amplicons containing segments within the promoter or coding region of the indicated genes were amplified by genomic PCR and purified. Each sequence was analyzed by qPCR using 1E-2, 1E-3, 1E-4, and 1E-5 nM template concentrations. The efficiency (E) of each primer set is shown.

Supplementary Figure S5. *cadR/cadA* promoter analysis for *in vitro* transcription. **(A – B)** *In vitro* transcription reactions were performed containing 0.1 U/µL *E. coli* RNA polymerase holoenzyme, 100 nM of the indicated *cadA/cadR* promoter template, 4 µM *Pa*CadR, and 100 µM $CdCl_2$. Samples were treated with DNase I and separated by denaturing PAGE. RNA was visualized by SYBR Gold

staining. RNA transcripts from *cadA* or *cadR* are annotated based on changes in corresponding template DNA lengths.

Supplementary Figure S6. *Pa*CadR binds DNA as a dimer *in vitro.* **(A)** DNA samples of various sizes and an EMSA reaction containing *Pa*CadR bound to its consensus sequence were separated by 5%, 6%, or 7% native PAGE. **(B)** Graph of the relationship between relative mobility (R_f) and acrylamide percentage. Relative mobility was determined by dividing the distance each band migrated through the gel by the distance migrated by the Orange G load dye in each sample. Linear regressions of the indicated protein samples are presented. **(C)** Graph of the negative slope from the regression models determined in (*B*) and apparent molecular weight. The apparent weight for the DNA-*Pa*CadR complex was identified using the presented linear regression model. The estimated weight of the *Pa*CadR consensus sequence is ~42 kDa. Therefore, the apparent weight of DNA-bound *Pa*CadR is ~38 kDa.

Supplementary Figure S7. *Pa*CadR exists as a dimer in solution. **(A)** Approximately 2 µg *Pa*CadR, *Pa*Ivyp1, or BSA were separated by 6% or 9% native PAGE. Proteins were visualized by Coomassie staining and gels were imaged using a LI-COR Odyssey imager. **(B)** Graph of the relationship between relative mobility (R_f) and acrylamide percentage. Relative mobility was determined by dividing the distance each band migrated through the gel by the distance migrated by the Orange G load dye in each sample. Linear regressions of the indicated protein samples are presented. **(C)** Graph of the negative slope from the regression models determined in (*B*) and apparent molecular weight. The apparent weight for *Pa*CadR was identified using the presented linear regression model.

Supplementary Figure S8. Further analyses of potential genomic binding sequences. IRDye-700 labeled genomic sequences from Table 1 (red) and IRDye-800 labeled control DNA (green) were incubated with 20, 40, 80, or 160 nM *Pa*CadR. Samples were analyzed by native PAGE and visualized using a LICOR Odyssey imager. Protein-bound (Bound) and unbound (Free) DNA complexes are identified. (*), nonspecific IRDye-800 PCR product.

Supplementary Figure S9. Expression of His-tagged CadR. **(A – B)** *P. aeruginosa* PAO1 strains containing the pME6001 replicative plasmid or a modified version of pME6001 containing the coding sequence for a His-tagged CadR construct were grown overnight in 40 mL LB, then lysed by sonication. Soluble fractions were incubated with Ni-NTA magnetic beads (New England Biolabs), washed, and then eluted in 2x Laemmli buffer. Samples were taken before incubating with the Ni-NTA beads (INPUT), from the lysate after Ni-NTA incubation (Supe), and from the eluted beads (IMAC Pur). These samples were separated by SDS-PAGE and visualized by Western blotting (*A*) and Coomassie staining (*B*). For the Western blot, samples were transferred to a PVDF membrane (Thermo Scientific), blocked with 5% nonfat milk in 1xTBST, incubated overnight with anti-His antibodies (Invitrogen RM146; 1:1000 dilution in 1xTBST with 1% BSA), washed in 1xTBST, and incubated with donkey anti-rabbit HRP antibodies (Invitrogen A16035; 1:100,000 dilution in 1xTBST with 1% BSA). Membranes were treated with SuperSignal West Fempto enhanced chemiluminescent substrate (Thermo Scientific) and visualized using a Bio-Rad ChemiDoc system.

Supplementary Figure S10. *cadA* promoter identification *in silico*. **(A)** Characterization of the *cadR* and *cadA* promoter region as shown in Figure 3B. **(B)** Sequences used as input into promoter identification software. The *Pa*CadR binding sequence is underlined. One, two or three nucleotides were removed from the middle of the *Pa*CadR binding sequence ("-1", "-2" and "-3", respectively).

(C) Identification of predicted *Pseudomonas* promoters from the indicated sequences using SAPPHIRE.CNN.pseudomonas. (Strand) promoter sequences were found on submitted DNA sequence (+) or complementary sequence (-). (Estimated TSS) the predicted transcription start site of the indicated promoter.

Supplementary Figure S11. Controls for Figure 4D. **(A – B)** RNA was isolated from various *P. aeruginosa* PAO1 strains after a 10-minute treatment with either 100 μM $CdCl_2$ or 100 μM $ZnSO_4$. Gene expression for *cadA* (*A*) or *cadR* (*B*) was quantified by RT-qPCR and normalized to the expression of *aceE* (pyruvate dehydrogenase E1 component, *PA5015*). Values are relative to the reference strain containing the empty pME6001 vector (pME6001-EV). Error bars represent one standard deviation between two independent experiments. A student's two-tailed *t*-test with unequal variance was used. p-value > 0.05 (n.s.), < 0.05 (*), < 0.005 (**).

Supplementary Figure S12: Evolutionary comparison of CadR binding sequences. Collection of CadR binding sequences from RegPrecise. Each binding motif resembles a 21 bp inverted repeat consisting of 10 bp repeat regions separated by one bp. Each repeat region is highlighted by the dashed box.
